# In this month’s Bulletin

**DOI:** 10.2471/BLT.19.000519

**Published:** 2019-05-01

In the editorial section, Flavio Salio and Altaf Musani (310) call for more research on the delivery of health services to people living in conflict settings. Mercedes Tatay and Els Torreele (311) ask for financing mechanisms to ensure access to essential medicines when countries are no longer eligible for assistance from the Global Fund.

Gary Humphreys (314–315) reports on an initiative to improve emergency care in district hospitals in Uganda. Rosalinda Dimapilis-Baldoz talks to Fiona Fleck (316–317) about the health worker migration policy of the Philippines and the *WHO Global code of practice on the international recruitment of health personnel*. 

## Afghanistan

### Adapting to an epidemiological transition

Karl Blanchet et al. (374–376) report on priority-setting measures. 

## Bosnia and Herzegovina

### What are people eating?

Selma Gicevic et al. (349–357) propose a low-cost model for dietary surveillance. 

## Hungary

### Funding social health insurance

Szabolcs Szigeti et al. (335–348) analyse revenue sources. 

## India

### Coping with shortages of poliovirus vaccine

Pradeep Haldar et al. (328–334) recount lessons learnt from fractional dosing. 

## Uganda

### How many people have chronic respiratory disease?

Trishul Siddharthan et al. (318–327) measure prevalence in urban and rural settings.

## United Republic of Tanzania

### Improving care during childbirth

Nanna Maaløe et al. (365–370) adapt intrapartum care guidelines. 

## Global

### Ensuring security of supply

David Beran et al. (358–364) assess vulnerabilities to insulin shortages.

### Supporting the trauma response 

Barclay Stewart et al. (371–373) argue that organized trauma care needs a greater share of development aid.

